# High-Intensity Intermittent Exercise Performed on the Sand Induces Higher Internal Load Demands in Soccer Players

**DOI:** 10.3389/fpsyg.2021.713106

**Published:** 2021-07-30

**Authors:** Tiago Cetolin, Anderson Santiago Teixeira, Juliano Fernandes da Silva, Alessandro Haupenthal, Fábio Yuzo Nakamura, Carlo Castagna, Luiz Guilherme Antonacci Guglielmo

**Affiliations:** ^1^Research Group for Development of Football and Futsal, Sports Center, Federal University of Santa Catarina, Florianópolis, Brazil; ^2^Physical Effort Laboratory, Sports Center, Federal University of Santa Catarina, Florianópolis, Brazil; ^3^Department of Health Sciences, Federal University of Santa Catarina, Araranguá, Brazil; ^4^Associate Graduate Program in Physical Education University of Pernambuco (UPE)/Federal University of Paraiba (UFPB), João Pessoa, Brazil; ^5^Research Centre in Sports Sciences, Health Sciences and Human Development (CIDESD), University Institute of Maia, ISMAI, Maia, Portugal; ^6^Fitness Training and Biomechanics Laboratory, Italian Football Federation (FIGC), Technical Department, Florence, Italy; ^7^Clinical Sciences and Translational Medicine Department, Faculty of Medicine, University of Rome Tor Vergata, Rome, Italy

**Keywords:** intermittent exercise, lactate response, maximal aerobic speed, maximal oxygen uptake (VO) 2 max, training surface

## Abstract

This study aimed to examine the acute physiological effect of shuttle-run-based high-intensity intermittent exercise (HIIE) performed at the same relative speed (i. e., 100% PS_T−CAR_) on sand (SAND) and grass (GRASS) in male junior soccer players. Seven Under-23 Brazilian national league (“Série A”) soccer players completed four testing sessions in either SAND or GRASS surface condition. The first two testing sessions consisted of performing a maximal progressive shuttle-run field protocol until volitional exhaustion (Carminatti's test, T-CAR), whereas the third and fourth sessions comprised a HIIE session on each ground surface. The HIIE session consisted of three 5-min bouts [12 s shuttle-run (with a direction change every 6 s)/12 s of passive rest] performed at 100% of T-CAR peak speed (PS_T−CAR_) with 3 min of passive recovery between sets. Measurements of oxygen uptake (VO_2_), heart rate (HR), blood lactate concentration ([La]), and rating of perceived exertion (RPE) were performed during all conditions. The SAND condition elicited significantly higher %VO_2_peak (94.58 ± 2.73 vs. 87.45 ± 3.31%, *p* < 0.001, *d* = 2.35), %HRpeak (93.89 ± 2.63 vs. 90.31 ± 2.87%, *p* < 0.001, *d* = 1.30), RPE (8.00 ± 0.91 vs. 4.95 ± 1.23 a.u., *p* < 0.001, *d* = 2.82), and [La] (10.76 ± 2.37 vs. 5.48 ± 1.13 mmol/L, *p* < 0.010, *d* = 2.84). This study showed that higher internal workloads are experienced by the players during a single HIIE session performed on a softer surface as SAND, even when the exercise intensity was individualized based on 100%PS_T−CAR_.

## Introduction

Shuttle-run-based high-intensity intermittent exercises (HIIE) with short intervals is considered as one of the most common forms of interval training in soccer (Dupont et al., 2004; Buchheit and Laursen, 2013a; Da Silva et al., 2015). Shuttle-run-based HIIE with short intervals consists of performing repeated running bouts lasting <45–60 s at an exercise intensity ranging from 100 to 120% of maximal aerobic speed interspersed with periods of rest or lower-intensity active recovery (Buchheit and Laursen, 2013a). Short-interval HIIEs are proposed with the aim to improve a range of soccer performance-related physical fitness attributes, such as aerobic fitness, intermittent running capacity, and repeated sprint ability (Dupont et al., 2004; Buchheit et al., 2008; Da Silva et al., 2015). In addition to the several exercise variables (i.e., work-to-rest ratio, recovery and work intensity, and training volume) (Buchheit and Laursen, 2013a), ground surface is also found to affect HIIE training outcome (Binnie et al., 2014a). Indeed, sand surface was proposed as an alternative training tool to improve relevant fitness components in team sports (Binnie et al., 2013a,b, 2014b). Despite low terrain specificity compared with natural grass and artificial turf, sand training was proposed with the aim to promote high internal load with drills supposedly reporting lower external loads (Binnie et al., 2013a,b, 2014b). Although sand training has gained popularity, studies examining the acute responses of shuttle-run-based HIIE models on cardiorespiratory, metabolic, and perceptual measures in soccer players on this softer surface are lacking, leaving the application of sand surface HIIE sessions speculative in this sport domain.

The effectiveness of different sand training methods not involving shuttle-run-based HIIE models (sport-specific drills, agility, and plyometrics) has been previously reported (Impellizzeri et al., 2008; Binnie et al., 2014a; Ramirez-Campillo et al., 2020). For instance, Binnie et al. (2014a) showed that sand training-induced larger gains in peak oxygen consumption (VO_2_peak) than grass training in team-sport players. Ramirez-Campillo et al. (2020) showed that a multi-surface (including sand) plyometric training led to greater improvements in neuromuscular performance compared with grass surface training in soccer players. A recent systematic review with meta-analysis also showed that sand training programs are able to induce positive changes in neuromuscular performance in team-sport players and that both training surfaces (sand and grass) are equally effective to improve sprint and jump performances (Pereira et al., 2021). Nevertheless, it should be noted that training exclusively on the sand was revealed to be detrimental to stretch-shortening cycle development, but tended to improve squat jump height more than grass surface (Impellizzeri et al., 2008). In view of the current evidence, coaches and practitioners can consider sand surface training as a suitable and effective means to enhance aerobic function and power-speed-related capacities in soccer and other team-sport athletes.

There are distinct psychophysiological and biomechanical requirements when running on sand and grass surfaces (Zamparo et al., 1992; Pinnington and Dawson, 2001a,b; Pinnington et al., 2005; Gaudino et al., 2013). Sand-based exercises elicit a higher oxygen uptake (VO_2_), heart rate (HR), rating perceived exertion (RPE), blood lactate concentration [La], and muscle activation pattern than grass surface exercises at similar speeds (Zamparo et al., 1992; Pinnington and Dawson, 2001a,b; Pinnington et al., 2005; Gaudino et al., 2013). Sand training also reported to induce lower exercise-induced muscle damage, soreness, and associated negative side effects (e.g., reduced performance), thereby demonstrating a decreased neuromuscular strain (Miyama and Nosaka, 2004). In addition, some changes in kinematic parameters (e.g., decrements in sprint speed and stride length) when running on sand (Pinnington et al., 2005; Alcaraz et al., 2011; Gaudino et al., 2013) has been similar to those observed when performing resisted sprints on grass surface using loads inferior to 20% of the body mass of the athletes (Pereira et al., 2021). Therefore, the compliant nature and unstable characteristics of sand could serve as a practical way to increase overload during workouts, without the need for using additional resistance or supplementary equipment (e.g., elastic bands) (Pereira et al., 2021). This information has relevance for training load prescription optimization across the soccer competitive season. From a practical perspective, replacing some grass training activities for sand-based exercises may be more indicated during the preseason phase (e.g., strength-oriented phase) (Binnie et al., 2014b; Pereira et al., 2021).

To date, the majority of the available studies reporting a greater SAND vs. GRASS internal training load considered only acute physiological responses during drills performed at similar running speeds (Zamparo et al., 1992; Lejeune et al., 1998; Pinnington and Dawson, 2001a,b). Furthermore, these studies used submaximal intensities ranging from 3 to 11 km/h, with the exception of three studies that involved sprint- and sport-specific drill (repeated sprint, agility, power exercises) sessions performed at a maximum perceived intensity (Binnie et al., 2013a,b; Gaudino et al., 2013). According to Binnie et al. (2014b), there is little evidence reporting the energy cost (EC) of running on sand at running speeds > 11 km/h. Similarly, studies comparing VO_2_ response during HIIE models performed at the same relative exercise intensity [e.g., running at 100% of maximal aerobic speed (MAS)] on sand and grass training surfaces are also unknown. The time spent at or near VO_2_peak—that is, ≥90% of VO_2_peak—has been considered a key criterion to define the effectiveness of training stimulus to improve VO_2_peak and aerobic running performance in soccer (Buchheit and Laursen, 2013a).

The final speed reached during maximal progressive field tests has been proposed as a valid metric to guide training prescription during shuttle-run-based HIIE sessions (Buchheit, 2008; Buchheit and Laursen, 2013a; Da Silva et al., 2015). More recently, the peak speed derived from Carminatti's test (PS_T−CAR_) provided a valid estimate of MAS in soccer players and proved to be an accurate reference speed for individualizing running distance (i.e., training intensity) during HIIE models implemented on grass surfaces (Da Silva et al., 2015). However, the acute physiological responses during a HIIE model performed at 100% PS_T−CAR_ on sand surfaces have not received attention to date. Considering that sand-based training has been used as a complementary strategy to promote variability in the training stimulus in team-sport players (Ramirez-Campillo et al., 2020; Pereira et al., 2021), it is of practical relevance to gather evidence regarding the accuracy of the PS_T−CAR_ for HIIE programming on this specific type of surface.

Thus, the present study examined the acute physiological effect of a single shuttle-run-based HIIE session performed at the same relative speed (i.e., 100% PS_T−CAR_) on sand and grass in male junior soccer players. As a hypothesis of this work, we assumed similar physiological responses across sand and grass HIIE sessions due to the individualization of training intensity across conditions.

## Materials and Methods

### Experimental Approach to the Problem

A randomized repeated-measures design was implemented. All participants were required to complete four testing sessions on two ground surfaces (SAND and GRASS) of distinct compliance/compactness to compare the acute physiological responses between the conditions tested. The testing sessions were carried out at the same time of the day and separated by at least 48 h to minimize any residual fatigue. The first two testing sessions consisted of performing a maximal progressive shuttle-run field test protocol until volitional exhaustion (T-CAR) on each ground surface to determine the relative peak values for the following variables: (i) speed achieved at the end of the T-CAR (PS_T−CAR_), (ii) heart rate (HR), (iii) blood lactate concentration ([La]), and (iv) RPE. Measurement of peak oxygen uptake (VO_2_peak) during the T-CAR protocol was obtained only on the grass surface. The third and fourth sessions comprised a HIIE model performed at 100% PS_T−CAR_ consisting of three sets of 5-min bout [12 s shuttle-run (with a direction change every 6 s)/12 s of passive rest] interspersed with 3 min of passive recovery between sets. Cardiorespiratory (given by VO_2_ and HR), metabolic (given by [La]), and perceptual (given by RPE score) responses were measured during the HIIE performed on each ground surface. The air temperature and relative humidity were as follows: T-CAR protocol on GRASS (26.0 ± 3.2°C and 62.1 ± 9.9%) and SAND (25.7 ± 4.4°C and 62.5 ± 9.3%); HIIE model on GRASS (26.6 ± 2.7°C and 53.8 ± 9.7%) and SAND (28.5 ± 2.3°C and 58.6 ± 7.3%).

### Participants

The sample size was previously estimated to induce a meaningful detectable effect size (Cohen's *d*) of 0.60 between training surfaces with the assumption of a statistical power of 0.90 at an alpha level of 0.05. The effect size used to generate the sample size was derived from previous investigations comparing the physiological responses between training surfaces assuming HR and RPE measures as references (Pinnington and Dawson, 2001b; Binnie et al., 2013a). The calculations were made using a customized computer software (GPOWER Version 3.1.9.2, Department of Psychology, Bonn University, Bonn, Germany). The analysis suggested a minimum sample of six players. Thus, nine male junior soccer players recruited from Under-23 team of a professional club competing in the Brazilian national league (“Série A”) took part in this study. Two players did not complete all testing sessions required for this study. Thus, data from only seven players (age: 18.37 ± 2.32 years; body mass: 65.95 ± 5.51 kg; height: 174.27 ± 6.84 cm; body fat: 10.65 ± 1.65%) were considered. At the time of the investigation (summer season), all the players were in their preseason training cycle. The current research proposal obtained ethical approval from the Local Research Ethics Committee of the University (n° 459.431). The club manager and parents or legal guardians of the participants were informed about the nature of the study including objectives, protocols, and related risks, and the participants provided informed written consent (>18 years) before the commencement of this study. Participation was voluntary and players provided assent (in the case of age < 18 years) after being informed that they could withdraw from the study at any time.

### Ground Surface Stiffness Determination

The GRASS testing session was conducted on a natural grass pitch (105 m of length and 68 m of width) at the club facilities, whereas the SAND testing session was performed in a training area with 30 m of length and 17 m of width. Surface stiffness for SAND and GRASS conditions was determined prior to each testing session using a Dynamic Cone Penetrometer (DCP) built by the civil engineering department of this University, weighing 8 kg and dropped from a height of 575 mm. On each occasion, 10 samples (spread over the entire training area) were taken to determine the ground stiffness [i.e., the depth of DCP penetration into the ground (mm)]. The ground stiffness was calculated as the mean value of the 10 samples obtained for each training surface. This technique has been detailed elsewhere (Davies and Mackinnon, 2006).

### Cardiorespiratory Measurements

During all the testing sessions, with the exception of the T-CAR protocol on the sand training surface, respiratory gas exchange was measured breath-by-breath using a portable system (Cosmed K4b^2^, Rome, Italy) to determine VO_2_ values. Before each testing session, the following K4b^2^ calibrations were performed: turbine flowmeter calibration (performed with a 3-L syringe, Quinton Instruments, Seattle, WA, United States), O_2_ and CO_2_ analysis systems (with a gas mix of 16% O_2_ and 5% CO_2_ concentrations), delay time, and ambient air calibration. The HR was recorded continuously during all testing sessions using a chest belt *via* short-range radio telemetry (Polar Team Sport System, Polar Electro Oy, Kempele, Finland).

The VO_2_ and HR data were reduced to 15 s mean values. The highest values obtained in this 15 s interval for VO_2_ and HR during the T-CAR protocol performed on the grass training surface were considered as VO_2_peak and HRpeak, respectively (Da Silva et al., 2015; Floriano et al., 2016). During the HIIE model on each training surface, the VO_2_ and HR data were reduced to 5 s mean values, and the VO_2_ and HR values obtained in each running set was considered as the average of the last two min of exercise (Floriano et al., 2016). The VO_2_ and HR responses for each running set (set 1, set 2, and set 3) were expressed as relative percentages of the VO_2_peak and HRpeak (%VO_2_peak and %HRpeak, respectively) reached in T-CAR.

### Blood Lactate Concentration

Capillary blood samples (25 μL) were collected by micropuncture at the ear lobe and then stored into microcentrifuge tubes containing 50 μL NaF (1%) in order to measure [La]. The analysis of blood lactate was performed using an electrochemical analyzer (YSI 2700 STAT, Yellow Springs, OH, United States). The [La] was collected 1 min after the cessation of each test (T-CAR protocol and each running set in both training surfaces).

### Rating of Perceived Exertion

The RPE of each player was assessed using the CR-10 scale proposed by Foster et al. (2001). All players were familiarized with the procedure before the study commencement. Players reported their RPE score immediately after the completion of the T-CAR and at the end of each HIIE set performed on both training surfaces.

### Carminatti's Test

The test consisted of intermittent shuttle-runs of 12 s performed between 2 lines set at progressive distances with a 6-s recovery time between each run and a total stage time of 90 s. The protocol had a starting velocity of 9 km/h over a running distance of 30 m (15 m back and forth). The length in a single direction was increased progressively by 1 m at every level. Each stage consisted of 5 repetitions of 12 s with a 6 s walking period between 2 lines set 2.5 m from the starting line. The running pace was dictated by a pre-recorded audio system (Da Silva et al., 2011; Teixeira et al., 2014). The test ended when participants failed to follow the audio cues on the front line for two successive repetitions (objective criteria observed by researchers). The last stage speed (PS_T−CAR_) during the T-CAR was retained to allow the individualization of the distance covered during the intermittent shuttle-run exercise protocol (12 s shuttle-run/12 s pause) used herein.

### High-Intensity Intermittent Exercise Session

Players completed a standardized warm-up consisting of a 5-min run at 50% of PS_T−CAR_ followed by 3 min of passive recovery before starting the HIIE model. Players performed three sets of 5-min bouts interspersed with 3 min of passive recovery between sets. Players performed repeated bouts of 12 s shuttle-runs (with a change of direction every 6 s) at 100% PS_T−CAR_ alternating with 12 s of passive rest until the completion of the 5 min established for each running set. The average running pace performed by the players between the start and return lines was dictated by a prerecorded audio cue, emitting beeps every 6 s. The distance covered by each player during the training sessions was individualized according to their respective 100% PS_T−CAR_.

### Statistical Analysis

Results are expressed as means ± SDs. After visual inspection, the Shapiro–Wilk test was used to verify the data normality. Levene's test was used to test if the homogeneity of variance was assumed. The inter-subject coefficient of variation (CV) was calculated for the %VO_2_peak, %HRpeak, [La], and RPE in each running set of the HIIE model. Paired Student's *t*-test was used to examine the differences between T-CAR protocols performed on the grass and sand training surfaces for HRpeak, [La], RPE, and ground stiffness. A mixed model analysis was used to compare acute physiological responses between the HIIE model conducted on each training surface (SAND and GRASS), assuming the training surface (SAND or GRASS) and running sets (set 1, set 2, and set 3) as fixed factors, and the participants as a random factor. When a significant F-value was obtained, a *post-hoc* test with a Bonferroni adjustment was performed for multiple comparisons. The magnitude of the differences was assessed using standardized mean differences (Cohen's *d* effect size, ES) with thresholds of 0.20, 0.60, 1.20, 2.0, and 4.0 for small, moderate, large, very large, and extremely large (Batterham and Hopkins, 2006). Statistical analyses were carried out using SPSS statistical analysis software (SPSS version 17.0, Chicago, IL, United States). The level of significance was set at *p* ≤ 0.05.

## Results

The depth of CPD penetration into the ground was significantly lower (*t* = −14.092, *p* < 0.001, *d* = 2.23) on GRASS (21.96 ± 10.53 mm) than for SAND surface (49.40 ± 13.90 mm), suggesting that the GRASS was a firmer surface than SAND.

After the completion of the T-CAR protocol, HRpeak (*t* = 0.236, *p* = 0.821, *d* = 0.16), [La] (*t* = −0.403, *p* = 0.701, *d* = 0.16) and RPE (*t* = −1.000, *p* = 0.356, *d* = 0.43) values did not differ between ground surfaces. The PS_T−CAR_ was significantly lower (Δ = −8.41 ± 6.21%, *t* = 3.745, *p* = 0.010, *d* = 1.58) on SAND than for GRASS surface (Table 1).

**Table 1:** Descriptive statistics (mean ± DP; and range) for physiological, metabolic, perceptual, and peak speed reached during the Carminatti's test performed on GRASS and SAND training surfaces.

	**GRASS**			**SAND**		
		**Range**			**Range**	
	**Mean ± DP**	**Min**	**Max**	**Mean ± DP**	**Min**	**Max**
VO_2_peak (mL/kg/min)	52.15, 2.89	47.94	55.67	n.a	n.a	n.a
Hrpeak (bpm)	198.0, 5.0	194.0	206.0	197.0, 8.0	191.0	208.0
PS_T−CAR_ (km/h)	15.23, 0.80^*^	14.00	16.20	13.89, 0.89	12.20	14.70
[La] (mmol/L)	8.85, 2.14	5.64	12.90	9.15, 1.59	7.17	12.00
RPE (a.u.)	8.00, 0.58	7	9	8.29, 0.76	7	9

*VO_2_peak, peak oxygen consumption; HRpeak, peak heart rate; PS_T−CAR_, peak speed reached at the end of Carminatti's test; [La], blood lactate concentration; RPE, rating of perceived exertion; n.a, not assigned*.

* *Significantly different from SAND surface (p = 0.010)*.

**Figure 1:**
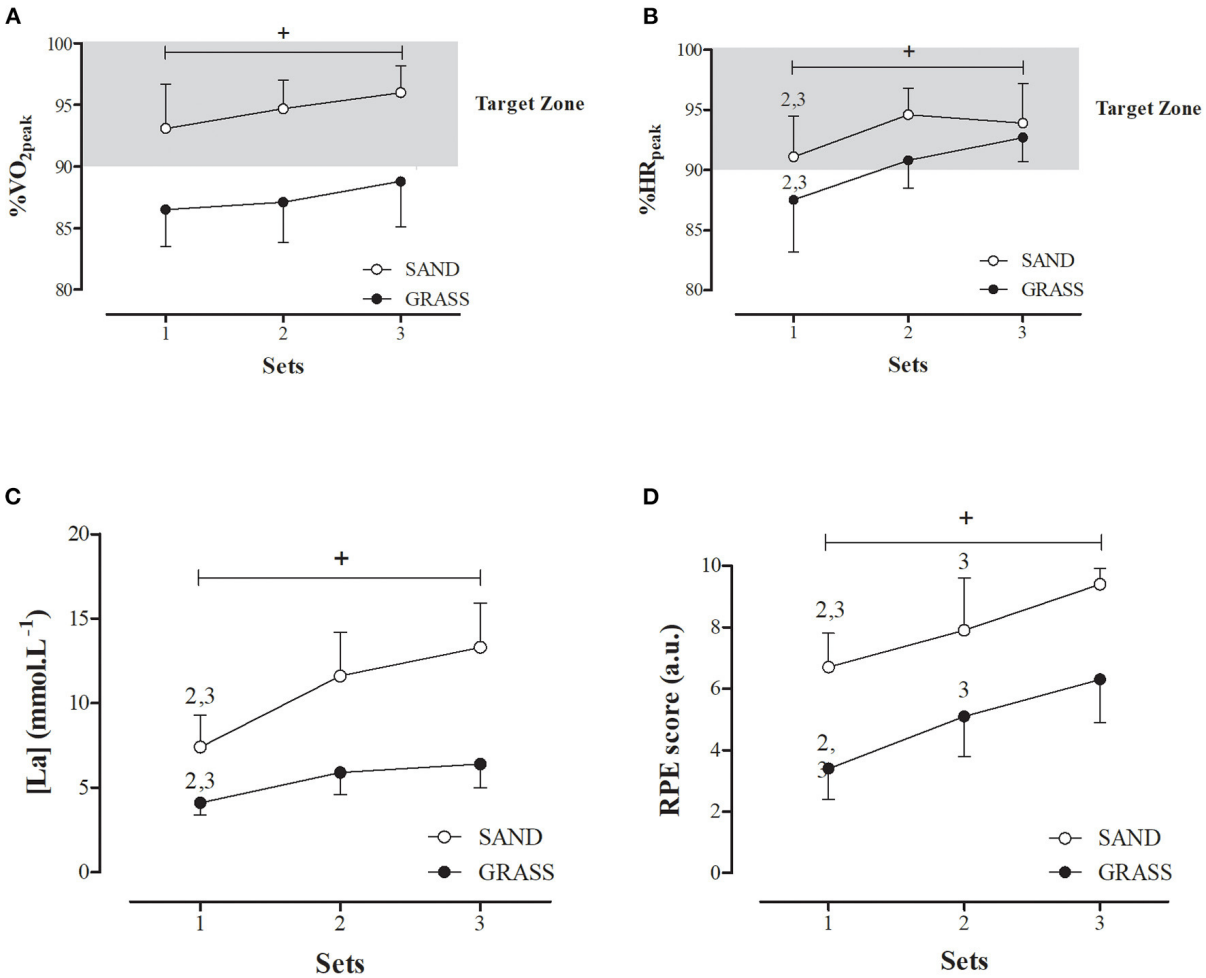
VO_2_ **(A)**, HR **(B)**, blood lactate [La] **(C)**, and RPE **(D)** responses to shuttle-run high-intensity intermittent exercise (HIIE) performed at 100% PS_T−CAR_ on SAND and GRASS surface condition. +: Denotes significant between-condition differences; 2: indicates a significant difference in relation to the second set; 3: indicates a significant difference in relation to the third set.

The analysis of HIIE did not reveal a significant condition-by-time interaction for %VO_2_peak (*F* = 0.099, *p* = 0.906), %HRpeak (*F* = 0.029, *p* = 0.971) and RPE score (*F* = 0.232, *p* = 0.795) nor a significant main effect of time (*F* = 2.440, *p* = 0.111) for %VO_2_peak. A significant main effect of condition (*F* = 56.592, *p* < 0.001, *d* = 2.29) was found for %VO_2_peak, with SAND eliciting a significantly higher %VO_2_peak than for GRASS (Figure 1A).

%HRpeak and RPE values were significantly influenced by the type of surface [%HRpeak (*F* = 16.239, *p* < 0.001, *d* = 1.04); RPE (*F* = 79.792, *p* < 0.001, *d* = 1.87)] and training sets [%HRpeak (*F* = 9.223, *p* = 0.001); RPE (*F* = 24.803; *p* < 0.001)]. *Post-hoc* tests identified that cardiovascular and perceptual responses (*p* < 0.001) were significantly higher on SAND than on GRASS. Compared with set 1, %HRpeak during set 2 and set 3 was significantly (*p* < 0.001) higher irrespective of the surface conditions. Differences across the sets were moderate to large (*d* = 1.22, 1.69, and 0.62) and moderate (*d* = 0.54, 0.86, and 0.88) for SAND and GRASS conditions, respectively (Figure 1B). The RPE progressively increased over the sets in both surfaces (Set 1 < Set 2 < Set 3). The resulting ES ranged from moderate to very large (*d* = 0.83–3.16 and *d* = 0.89–2.38 for SAND and GRASS, respectively) (Figure 1D).

There was a significant condition-by-time interaction effect for [La] (*F* = 4.110, *p* = 0.035) with significantly higher [La] values for the SAND condition (set 1: *p* = 0.003, *d* = 2.31; set 2: *p* = 0.001, *d* = 2.77; set 3: *p* < 0.001, *d* = 3.30). Increments in [La] from sets 1–2 [Δ = 4.2 ± 2.1 mmol/L (*p* = 0.017, *d* = 1.84) vs. Δ = 1.8 ± 0.9 mmol/L (*p* = 0.033, *d* = 1.72)] and sets 1–3 (Δ = 5.9 ± 2.7 mmol/L (*p* = 0.001, *d* = 2.59) vs. Δ = 2.3 ± 0.9 mmol/L (*p* = 0.011, *d* = 2.08)] were significantly greater on SAND than on GRASS surface, respectively (Figure 1C).

A low inter-subject CV for cardiorespiratory responses (VO_2_ and HR) was observed during the HIIE session performed on SAND (%VO_2_peak: 2.31–3.92%; %HRpeak: 2.31–3.78%) and GRASS (%VO_2_peak: 3.47–4.12%; %HRpeak: 2.18–4.91%). On the other hand, a higher inter-subject CV was noticed for metabolic and perceptual responses on SAND ([La]: 19.28–25.98%; RPE: 5.67–16.57%) and GRASS ([La]: 16.92–22.97%; RPE: 21.96–28.46%) conditions.

## Discussion

This is the first study that investigated the effects of SAND vs. GRASS training surfaces on internal load (i.e., VO_2_, HR, RPE, and [La]) during a HIIE session performed at 100% PS_T−CAR_ in junior male soccer players. The results showed that the SAND condition elicited significantly higher %VO_2_peak, %HRpeak, RPE, and [La] values than GRASS training surface, thus not confirming this study work hypothesis.

In the present study, the peak values of HR, [La], and RPE after the completion of T-CAR protocol did not differ between both training surfaces, with trivial to small ES (Table 1). On the other hand, PS_T−CAR_ was, on average, 8.4% slower on SAND than on GRASS surface. These findings are similar to those reported by Cetolin et al. (2010) who also demonstrated a lower PS_T−CAR_ (−7.0%) at the end of the T-CAR performed on SAND compared with GRASS surface. Running on SAND is characterized by a lower horizontal take-off velocity and a subsequent shorter forward distance traveled during the flight phase of the stride, which directly implies a reduced stride length, especially in the highest running speeds (Pinnington et al., 2005; Alcaraz et al., 2011; Gaudino et al., 2013). These movement pattern changes in association with a higher running cost may explain the observed lower PS_T−CAR_ in the SAND condition (Zamparo et al., 1992; Pinnington and Dawson, 2001a,b; Gaudino et al., 2013). Indeed, previous research showed that SAND demanded significantly higher VO_2_ and [La] values than running on GRASS at comparable speeds, thus resulting in a steeper slope in the VO_2_ and [La] vs. running speed relationship (Pinnington and Dawson, 2001b). However, possible inferences regarding the energy contribution during the T-CAR should be interpreted with caution, since neither the present nor the previous study of Cetolin et al. (2010) measured VO_2_ responses during submaximal T-CAR speeds on SAND surface. Thus, future studies should further elucidate the extent to which these aforementioned aspects and other potential factors (e.g., peripheral fatigue) contribute to the slower PS_T−CAR_ observed in SAND.

The reported significant and practically large differences in PS_T−CAR_ and the non-significant correlation (*r* = 0.382; *p* = 0.398) between GRASS and SAND conditions strongly suggest test condition specificity. This means that those players who perform better on GRASS will not necessarily be the ones who perform better on SAND. This finding has practical relevance for strength and conditioning coaches when programming HIIE sessions on different training surfaces. For instance, the use of PS_T−CAR_ determined on GRASS as the reference speed for prescription of sand-based HIIE protocols could be problematic, since players would be exercising at a supramaximal intensity and, in turn, the same set duration prescribed for GRASS surface could not be sustained by the players when running on SAND. In addition, the distinct relative external load experienced by the players could impose a divergent workload, contributing to a wider variability in the response of the athlete to the training stimulus. Interestingly, the use of surface-specific relative training speeds (i.e., PS_T−CAR_) resulted in a low inter-subject CV in the cardiorespiratory responses (VO_2_ and HR) during the HIIE protocol performed on both SAND and GRASS surfaces (see section Results). Thus, it was possible to ensure a similar internal training load to all the players in each training surface condition using PS_T−CAR_ as the reference speed for training prescription, at least while considering the cardiorespiratory responses. The inter-subject CV for [La] in our study was similar to that found by Julio et al. (2020) (CV: 18–28%) using the anaerobic speed reserve as a parameter to calibrate exercise intensity. These data highlight the applicability of PS_T−CAR_ as an accurate metric to individualize exercise intensity (i.e., running distance) during shuttle-run-based HIIE sessions in male soccer players.

The acute physiological responses during the HIIE model were surface-specific. Indeed, the average HR, VO_2_, RPE, and [La] values were 1.04-, 1.08-, 1.62-, and 1.96-fold higher, respectively, on SAND compared with those on GRASS. Moderate-to-large differences were found between surfaces for the physiological outcomes considered. Our findings are in agreement with those reported in other studies that also showed a higher demand in terms of cardiorespiratory, metabolic, and perceptual responses on SAND compared with GRASS using similar absolute running speeds (Pinnington and Dawson, 2001a,b; Binnie et al., 2013a,b, 2014b). Although the reasons for these aforementioned physiological, metabolic, and perceptual differences are not completely elucidated in the literature, some factors can aid to explain the findings of this study. From a biomechanical perspective, a more flexed hip and knee position associated with a greater muscle activation has been reported to support body mass and to stabilize lower limbs joints during the support phase of the stride when running on SAND (Pinnington et al., 2005). The resulting alteration in running kinematics and associated greater muscle recruitment may be considered as the possible cause of the greater cardiovascular and metabolic load (i.e., EC) of SAND running. The changes of direction of the sand-based HIIE model used here also deserve attention. Gaudino et al. (2013) found more intense maximum deceleration activities on SAND compared with those on GRASS surfaces during shuttle-sprint efforts. Thus, it could be speculated that the increased energetic demand verified on SAND might be, in part, related to the more intense deceleration movements required in our HIIE protocol performed on SAND. In addition, the greater muscle work when running on SAND (Pinnington et al., 2005) due to the longer foot contact time (Lejeune et al., 1998; Gaudino et al., 2013) may also have increased the EC to re-accelerate after each change of direction compared with the GRASS condition. Indeed, accelerating on SAND was reported to require a 30% extra EC compared with the GRASS condition (Gaudino et al., 2013). However, further studies are necessary to better understand the contribution of acceleration and deceleration movements to the increased energy demand during shuttle-run-based HIIE sessions when performed on SAND.

The time spent at intensities equal to or higher than 90% of VO_2_peak or HRpeak plays a pivotal role in the aerobic adaptive responses to training (Castagna et al., 2011, 2013). For this reason, the time spent at high intensity has been reported as the variables conditioning HIIE effectiveness (Buchheit and Laursen, 2013a). Interestingly, SAND promoted a higher permanence at or above the reported effective HIIE intensity. With regard to [La], the values over sets were classified as high to very high for SAND, and low for GRASS surface (Buchheit and Laursen, 2013b). Similarly, players perceived HIIE session as harder on SAND (RPE > 7.0 a.u.) than on GRASS surface (3.0 < RPE < 7.0 a.u.) Interestingly, the considered bout duration and work-to-rest ratio (i.e., 12 s and 1:1) were not effective to warrant values above 90% of VO_2_peak and HRpeak on GRASS condition (Figure 1). In an earlier study (Da Silva et al., 2015), players achieved HR values between 90 and 95% of HRpeak during the same HIIE protocol outlined here (12 s:12 s) when performed on GRASS surface, but considering four sets of 4 min. However, a slightly lower cardiovascular demand (85–90% of HRpeak) was observed with shorter running bouts (i.e., 6 s at 100% PS_T−CAR_) (Da Silva et al., 2015). Even so, the two HIIE formats (12 s:12 s or 6 s:6 s) proved to be an efficient training stimulus to improve some aerobic fitness measures (e.g., anaerobic threshold and running speed at VO_2_peak) and PS_T−CAR_ during the preseason phase in male junior soccer players (Da Silva et al., 2015). Nonetheless, changes in VO_2_peak were negligible in the cited study conducted by Da Silva et al. (2015). These results may be associated with the short duration of the training program (only 5 weeks), but also with the “low” VO_2_ demand (mean values below 90% VO_2_peak) on GRASS elicited by the proposed HIIE protocol (i.e., a work-to-rest ratio of 1:1).

Understanding that the training process is planned from an integrative and multicomponent perspective, coaches and sports scientists should also consider the potential of SAND-based training as a means of developing some power-speed-related capacities in team-sport players. A recent systematic review with meta-analysis showed that sand and hard surface training interventions were similarly effective at improving both jump and sprint performances in team-sport players (Pereira et al., 2021). The authors argue that these neuromuscular performance adaptations displayed in both surfaces are underlined by two distinct—but possibly complementary—mechanisms. While the performance adaptations provided by hard surfaces may be more related to the improvement in the stretch-shortening cycle efficiency, on sand surfaces they seem to be more attributable to changes in muscle contractile properties (Pereira et al., 2021). Taking into account the high neuromuscular load required during shuttle-run (Gaudino et al., 2013) along with the increased muscle activation level observed on sand surface (Pinnington et al., 2005), future studies are encouraged to investigate the potential effectiveness of sand-based HIIE programs involving shuttle-run on jump and speed performance in soccer players and other team-sport players.

Some limitations should be addressed. First, due to logistical reasons, respiratory gas exchange measures (e.g., VO_2_ response) were not evaluated during the T-CAR protocol performed on SAND, which prevented the comparisons of VO_2_peak and VO_2_-speed relationship between both training surfaces. In addition, submaximal responses of HR for the same running speed during T-CAR in both surfaces were also not recorded. Second, the lack of VO_2_ responses during T-CAR on SAND also limited the interpretation of VO_2_ demand during the HIIE in this specific surface, as the relative VO_2_ values achieved during the sand-based HIEE session were expressed in function of VO_2_peak observed on GRASS condition. Third, physical demand in terms of acceleration and deceleration movements during our HIIE protocol were not quantified. Fourth, despite the wide use of HR for soccer training prescription, it is important to recognize that this variable has some limitations for monitoring activities in an intermittent regime (Buchheit and Laursen, 2013a).

### Practical Applications

The originality of this study lies in addressing specific acute physiological responses (VO_2_, HR, [La], and RPE) to the training surface (SAND and GRASS) during a HIIE protocol performed at the same relative exercise intensity (i.e., 100% PS_T−CAR_) in male soccer players. Another strength of this study was the applicability of PS_T−CAR_ as an accurate metric to individualize exercise intensity (i.e., running distance) during the HIIE model in the SAND and GRASS training conditions. From a practical standpoint, strength and conditioning coaches should pay attention to why players did not achieve VO_2_ values above 90% of their individual VO_2_peak during the HIIE session performed on the GRASS surface. This suggests that some adjustments in training variables (e.g., increase work-to-rest ratio or running bout and set duration) may be necessary to allow a high percentage of time spent above 90% of VO_2_peak during HIIE session at 100%PS_T−CAR_ performed on the grass surface. Another suitable and practical alternative supported by this study is replacing the GRASS surface with SAND-based exercises. The sand-based HIIE session outlined here has a great potential to maximize gains in VO_2_peak and aerobic running performance in the initial phases of the season (i.e., preseason), since this training stimulus elicited a higher cardiorespiratory load and anaerobic glycolytic contribution. In addition, a prior research that compared several energetic and biomechanical parameters of sprinting between SAND and GRASS (natural and artificial) has suggested that shuttle-run exercises might be a better strategy to generate more intense deceleration actions in a softer surface, such as SAND (Gaudino et al., 2013). Given that soccer is a sport with a greater frequency of high- and very–high-intensity decelerations compared with accelerations (Harper et al., 2019), the presence or absence of changes of direction should be taken into account during the HIIE programming in order to manage the eccentric workload that will be experienced by the players in this type of activity performed on SAND.

## Conclusions

This study showed that the SAND surface elicited a higher internal workload in terms of cardiorespiratory, metabolic, and perceptual response than the GRASS surface during a single HIIE session performed at the same relative exercise intensity (i.e., 100%PS_T−CAR_). Furthermore, PS_T−CAR_ can be used as an accurate reference speed to individualize the running distance during HIIE with short intervals either in the SAND or in the GRASS condition.

## Data Availability Statement

The raw data supporting the conclusions of this article will be made available by the authors, without undue reservation.

## Ethics Statement

The studies involving human participants were reviewed and approved by The Research Ethics Committee of the Federal University of Santa Catarina (Number 459.431). Written informed consent to participate in this study was provided by the participants' legal guardian/next of kin.

## Author Contributions

TC, JS, and LG participated in the conception and design of the study. TC, JS, and AH participated in the data collection. AT, FN, and CC participated in the data analysis and interpretation. All authors participated sufficiently in the work drafting the study and/or revising it critically and approved the final version to be published.

## Conflict of Interest

The authors declare that the research was conducted in the absence of any commercial or financial relationships that could be construed as a potential conflict of interest.

## Publisher's Note

All claims expressed in this article are solely those of the authors and do not necessarily represent those of their affiliated organizations, or those of the publisher, the editors and the reviewers. Any product that may be evaluated in this article, or claim that may be made by its manufacturer, is not guaranteed or endorsed by the publisher.
